# Phosphodiesterase 10A Inhibition Leads to Brain Region-Specific Recovery Based on Stroke Type

**DOI:** 10.1007/s12975-020-00819-8

**Published:** 2020-05-06

**Authors:** Shirin Z. Birjandi, Nora Abduljawad, Shyama Nair, Morteza Dehghani, Kazunori Suzuki, Haruhide Kimura, S. Thomas Carmichael

**Affiliations:** Department of Neurology and of Neurobiology, David Geffen School of Medicine at UCLA, Los Angeles, CA; Department of Neurology and of Neurobiology, David Geffen School of Medicine at UCLA, Los Angeles, CA; Department of Psychology and of Computer Science, University of Southern California, Los Angeles, CA; Neuroscience Drug Discovery Unit, Research, Takeda Pharmaceutical Company Limited, Fujisawa, Kanagawa, Japan; Department of Neurology and of Neurobiology, David Geffen School of Medicine at UCLA, Los Angeles, CA

**Keywords:** repair, axonal sprouting, angiogenesis, BDNF, striatum

## Abstract

Stroke is the leading cause of adult disability. Recovery of function after stroke involves signaling events that are mediated by cAMP and cGMP pathways, such as axonal sprouting, neurogenesis, and synaptic plasticity. cAMP and cGMP are degraded by phosphodiesterases (PDEs), which are differentially expressed in brain regions. PDE10A is highly expressed in the basal ganglia/striatum. We tested a novel PDE10A inhibitor (TAK-063) for its effects on functional recovery. Stroke was produced in mice in the cortex or the striatum. Behavioral recovery was measured to 9 weeks. Tissue outcome measures included analysis of growth factor levels, angiogenesis, neurogenesis, gliogenesis and inflammation. TAK-063 improved motor recovery after striatal stroke in a dose-related manner, but not in cortical stroke. Recovery of motor function correlated with increases in striatal brain-derived neurotrophic factor. TAK-063 treatment also increased motor system axonal connections. Stroke affects distinct brain regions, with each comprising different cellular and molecular elements. Inhibition of PDE10A improved recovery of function after striatal but not cortical stroke, consistent with its brain localization. This experiment is the first demonstration of brain region-specific enhanced functional recovery after stroke, and indicates that differential molecular signaling between brain regions can be exploited to improve recovery based on stroke subtype.

## Introduction

Stroke is one of the leading causes of adult disability in the United States [1]. Neurorehabilitation after stroke leads to modest improvements in motor recovery [2], but there is currently no drug regimen that enhances recovery after stroke. Cellular and molecular mechanisms of recovery after brain injury involve changes in glial cell activation [3], alterations in cellular excitability [4, 5], and induction or delivery of a molecular signaling cascade that results in elevated growth factors, such as brain-derived neurotrophic factor (BDNF) [6, 7]. Recently, it has been shown that the transcription factor CREB, which induces BDNF, promotes recovery after stroke [8]. However, CREB is present in all cells and directly modulating CREB levels would be therapeutically difficult.

Phosphodiesterases (PDEs) degrade the intracellular second messengers cAMP and/or cGMP and terminate intracellular signaling that leads to CREB activation [9]. Clinically, PDE inhibitors are currently used for the treatment of acute heart failure [10] and erectile dysfunction [11]. Accumulating preclinical evidence indicates that PDE inhibitors promote learning and memory and attenuate disease progression in several neurologic illnesses [12, 13]. To date, eleven isoforms of PDEs have been identified [14]. Among these PDE families, PDE10A is unique in its abundance in the basal ganglia/striatum. Elevated levels of cAMP and/or cGMP in this brain lead to increases in BDNF [13]. The use of a PDE10A inhibitor has not been examined in recovery after stroke. It is possible that selective induction of BDNF in the striatum may enhance recovery in subcortical or striatal stroke, a common stroke subtype [15] but not have an effect in other stroke subtypes, such as in cortex or other brain areas in which PDE10A levels are low.

We hypothesized that a PDE10A inhibitor would promote recovery in striatal but not cortical stroke based on the high expression level of PDE10A in the striatum. We show that mice given a novel PDE10A inhibitor, TAK-063 [16, 17], have improved motor recovery after striatal stroke in a dose-dependent manner, but not cortical stroke. Recovery of motor function correlates with increases in striatal BDNF, downstream of the cAMP signaling cascade, and is associated with preservation in motor system connections and angiogenesis.

## Materials and Methods

### Animals

The National Institute of Health (NIH) guidelines for the care of laboratory animals were implemented. The study was approved by the IACUC (Institutional Animal Care and Use Committee) of the University of California Los Angeles (UCLA). Male 10-week old C57BL/6 mice weighing approximately 25g were used (Jackson Laboratories, (Bar Harbor, ME). Young adult male mice were used in these exploratory or discovery experiments, with later follow-up planned on aged and female mice. Animals were housed 4 per cage under pathogen-free conditions. Mice observed to be aggressive were separated as appropriate. Animals were housed in a light-controlled environment with a reverse light cycle (lights on from 12 AM to 12 PM) with free access to irradiated pellets (LabDiet PicoLab, Rodent Diet 20) and sterilized and acidified water. Housing temperatures were maintained between 20 to 21°C and standard humidity between 30 to 70%. For treatment, mice were injected once daily intraperitoneally (i.p.) with TAK-063 at 0.3 mg/kg, 3.0 mg/kg, and 10 mg/kg doses. 0.5% methylcellulose (MC) in sterile saline was used as the vehicle control. Full animal numbers and any animal exclusions are in Supplementary Methods.

#### 5-Ethynyl-2’deoxyuridine

5-Ethynyl-2’deoxyuridine (EdU) was dissolved in PBS at 5 mg/mL, filter sterilized, and injected i.p once daily at 25 mg/kg starting 2 days post-striatal stroke for 5 consecutive days [18]. 200 μg/mL EdU + enrofloxacin was further supplemented in rodent drinking water ad libitum 7 days post-striatal stroke. The water was changed every 48 hours and mice were exposed to EdU for 6 weeks. EdU was visualized using ClickiT labeling kit (Life Technologies) as detailed in the manufacture’s protocol.

### TAK-063 Preparation

TAK-063 (Takeda Pharmaceutical Company Limited, Fujisawa, Japan) was prepared in 0.5% (w/v) methylcellulose (MC) in sterile saline. Briefly, 5 g MC (METOLOSE® SM-100, Shinetsu Chemical Co. Ltd., Japan) was dissolved in 1 L of sterile saline. The solution was allowed to mix for several hours at room temperature until completely dissolved. Prepared MC saline was then placed at 4°C. A stock solution of 5 mg/mL TAK-063 was made fresh twice weekly by placing 5 mg of compound into 1 mL of MC saline. The solution was then vortexed and sonicated for 20 minutes in an ultrasonic bath. Dilutions for the above treatment groups of the 5 mg/mL stock solution were done using 0.5% MC saline. Diluted compound was sonicated for 10 minutes before daily drug injection. The drugs were administered after the completion of behavioral tests each day.

### Stroke Production:

#### Striatal Stroke

Ten week old adult male C57BL/6 mice (Jackson Labs) were placed under 2.5% isoflurane anesthesia and their necks were shaved and aseptically prepared. The left common carotid artery was exposed through a ventral midline incision in the neck, and the artery was gently separated from the carotid sheath and vagus nerve and permanently occluded by cauterization. Mice were subsequently placed on a stereotaxic apparatus and their heads were shaved and swabbed with alcohol and betadine. Their skulls were then exposed using a midline incision and cleared of connective tissue. A single burr hole was made in the skull (0.95 mm anterior, 2.3 mm lateral to Bregma) and the skull was cooled using a cotton swab soaked in saline. A needle was angled at 10° over the burr hole and inserted 3.0 mm down from the surface of the brain. After one minute, the needle was raised to 2.6 mm and 3 μL of L-NIO solution (27 mg/mL) was infused at 0.3 μL/minute using the Hamilton automated injection apparatus. Five minutes after infusion, the needle was raised to 2.2 mm from the brain surface and then subsequently raised again to 0.2 mm. After an additional minute, the needle was slowly retracted from the brain, the skull was rinsed using sterile solution, and the incision closed. The temperature of all mice was monitored with a rectal probe and maintained at 37°C (±5°C) with a heating pad. After surgery, the mice were allowed to recover and regain consciousness and then returned to their home cage. Mice that did not survive surgery were excluded from the study.

#### Cortical Stroke

Under isoflurane anesthesia (2–2.5% in a 70% N_2_O/30% O_2_ mixture), 10-week-old male C57BL/6 (Jackson Labs) mice were placed in a stereotactic apparatus; their skull was exposed through a midline incision, cleared of connective tissue, and dried. A cold light source (KL1500 LCD, Zeiss) attached to a 40× objective giving a 2-mm diameter illumination was positioned 1.5 mm lateral from the bregma, and 0.2 mL of Rose Bengal solution (Sigma; 10 g/L in normal saline, i.p.) was administered. After 5 minutes, the brain was illuminated through the intact skull for 15 minutes. Rose Bengal produces oxygen radicals under light excitation, occluding vascular endothelium, thus resulting in focal cortical stroke under the region of illumination.

### BDA Injections

Animals were injected with the neuroanatomical tracer 10% biotinylated dextran amine (BDA) (10,000 MW; Invitrogen) into the motor cortex ipsilateral to the site of stroke 9 weeks post stroke. Briefly, mice were anaesthetized and prepared in the stereotaxic apparatus and a burr hole was made (1.75 mm anterior, 1.5 mm lateral Bregma). A needle was placed at 90° over the burr hole and inserted 1.2 mm from the surface of the brain. The needle was raised to 1.0 mm after 1 minute and 0.3 μL of BDA was infused at 0.05 μL/minute using the Hamilton automated injection apparatus. After 6 minutes, infusion was complete and the needle was slowly raised to 0.2 mm from the surface of the brain and an additional minute was allowed to prevent the withdrawal of BDA through capillary action. The needle was then removed and the incision was closed. As before, body temperature of all mice was maintained and they were allowed to recover and return to their home cage. One week following BDA injections mice were euthanized. Analysis of BDA-positive fibers was performed using ImageJ. 20× magnification; sections were imaged via a confocal microscope (Nikon C2). The integrated density, corresponding to mean gray value multiplied by area of the brain analyzed (such as ipsilateral or contralateral striatum), was determined for each animal. The mean integrated density across three sections was calculated and reported by investigators *blind* to all treatment groups. Normalization was performed to each animal’s site of BDA injection. Background integrated densities were measured for each section at the site of BDA injection and subtracted from integrated densities obtained for either ipsilateral or contralateral striatum. Mice that did not survive BDA surgery were excluded from the study. In addition, mice that did not take up the BDA to label axons were excluded from the BDA analysis studies.

### Behavioral Testing

#### Grid-Walking Task

A baseline test was first conducted 7 days before surgery and subsequently at 1, 3, 6 and 9 weeks post stroke. Each mouse was tested once on each week’s testing day at approximately the same time during the dark cycle. The grid-walking apparatus was constructed using a box with a base made of metal wire mesh with 1 cm by 1 cm square openings. The grid area was 32 cm by 20 cm and the box was suspended 24 inches above a mirror. A video camera was placed underneath the grid to record the reflection of mice performing the task and to allow assessment of stepping errors (foot faults). Each mouse was placed on top of the grid and allowed a period of 5 minutes to walk freely as described by Clarkson et al. [5, 4]. The video footage was subsequently analyzed offline for the total number of steps taken and the total number of foot faults by the right forelimb (contralateral to the stroke site); evaluators were blinded to the treatment groups. A step was defined as the forward movement of all 4 paws beginning with a forepaw and ending with a hind paw, while a foot fault was defined as the passage of an entire paw through the plane of the wire. To assess deficit/recovery in gait and control after experimental stroke, the percentage of foot faults was calculated using the following equation: number of right forelimb foot faults/total number of steps × 100. This percentage value was obtained at baseline and for 1, 3, 6 and 9 weeks post-stroke. A normalized value was then obtained by subtracting the % right faults at baseline from the % right faults at each post-stroke evaluation. This test has repeatedly correlated with treatments that improve behavioral recovery after stroke, with low variance animal-to-animal and high reproducibility in findings across studies [19, 5, 4, 8, 20].

#### Forelimb Task (Cylinder Task)

This task allows the assessment of forelimb use during the exploration of the vertical walls of a cylinder [21]. This cylinder was high enough that rearing by the mouse would not enable it to reach the top edge and wide enough to allow space for exploratory behavior (height = 15 cm, diameter = 10 cm). Similar to studies by Baskin et al. [22], animals were placed in the cylinder and videotaped for 5 minutes. Two mirrors were placed at angles vertically behind the cylinder to allow the video camera to capture rears in the opposite plane of view. Footage was subsequently quantitatively analyzed to determine forelimb preference during exploratory vertical movement by investigators blind to all treatment groups. Using the ‘contact placement’ method described by Schallert et al [21], video footage was played in slow motion (1/5^th^ real time speed) and the number of rears using the right forelimb (ipsilateral to the lesion), the left forelimb (contralateral to the lesion), or both forelimbs was determined. The placement of a second forelimb within 3 frames of the first was counted as a “both” rear and only rears in which both forelimbs were clearly visible were counted. The number of right forelimb rears was analyzed to establish the level of use of only the impaired forelimb and was normalized to the animal’s baseline value.

### Statistics

Investigators blinded to the treatment groups performed behavioral analyses. All values in the figures are presented as mean ± Standard Error of the Mean (SEM). Behavioral data were tested with general linear model (GLM) with Tukey’s HSD (honest significant difference) using the software package R 3.1.3. Differences at *P* ≤ 0.05 were considered statistically significant. The sample sizes for each group were determined from power analysis from a study of a gamma-aminobutyric acid (GABA) Aα receptor negative allosteric modulator in stroke recovery [5]. Based on the assumptions of alpha = 0.05 and power = 0.080, the estimated sample size for a greater than 2 group comparison is 6.7 mice per group. Animals were assigned to randomly to experimental groups using True Random Number Generator software (Randomness and Integrity Services Ltd). A cube-root transformation was performed on the dependent variable in each dataset, due to either non-normality or non-heterogeneity of the variance. After each transformation, a Levene’s test was performed to assess the differences between the variances. The transformed data was also visually inspected, confirming that the data is approximately normally distributed, with a peak in the middle and fairly symmetrical. Therefore, we continued with a parametric test for each dataset. Differences between two means were assessed by unpaired two-tailed Student’s *t* test for BDNF data, p values were corrected for multiple comparisons, and differences among multiple means for axonal sprouting, IHC tissue, and Nissl-stained sections were assessed by one-way analysis of variance (ANOVA) followed by Tukey-Kramer’s *post hoc* tests.

### Tissue Fixation and Staining

Seven days after BDA injection, animals were euthanized with 100% isoflurane and perfused through the left ventricle with 0.1 mol/L phosphate buffered saline (PBS) followed by 4% paraformaldehyde (PFA) at 4°C. Brains were removed and subsequently fixed in 4% PFA overnight at 4°C. Brains were then moved to a 20% sucrose solution for cryoprotection. After 2 days, all tissue was removed and brains were rapidly frozen using dry ice and then stored at −80°C. All brains were later cut at 50 μm using a cryostat and four series of coronal sections, each spaced 300 μm apart were collected and placed in wells containing antifreeze solution. A single series was later removed from antifreeze, rinsed in 1× PBS and mounted on subbed glass slides.

### Nissl Staining

Slides were then run through a series of solutions to stain for cell bodies using the Nissl staining procedure: slides were first run through ascending alcohol solution (50%, 75%, 95% and 100%) and then placed in a 1:1 alcohol/chloroform solution for 30 minutes to de-fat tissue sections. Sections were then rehydrated in descending alcohols, rinsed in distilled water, and stained in cresyl violet solution for 1 minute. Slides were removed from the solution after this period and rinsed in distilled water and then dehydrated in ascending alcohol solutions. This de-staining step was carefully monitored to ensure visibility of all structures. Finally, sections were cleared in xylenes and cover slipped.

### Infarct Analysis

Using StereoInvestigator quantification software (MBF Bioscience), the total hemispheres ipsilateral and contralateral to the infarct from cresyl violet-stained sections were traced by investigators blind to all treatment groups and a value obtained. Results were reported as a ratio of ipsilateral/contralateral. For the striatal stroke tracing was divided into four zones (Zone A-D). With Zone A at 0.80 mm to 50 mm, Zone B at 0.26 mm to −0.10 mm, Zone C at −0.22mm to −0.82mm, and Zone D −1.22 mm to −1.94 mm from Bregma). The cortical strokes were measured from 1.10 mm to −2.06 mm from Bregma. Averages of the traced sections were grouped and obtained. Animals with absence of infarct were excluded from the study.

### Immunohistochemistry

Fluorescence immunohistochemistry with floating frozen sections was performed as previously described [23]. Briefly, primary antibodies were as follows: rat anti-GFAP (Invitrogen); goat anti-IBA-1 (Abcam); rat anti-Glut-1 (Abcam); rabbit anti-Olig2, (Millipore); rabbit anti-NeuN, (Abcam); and rat anti-CD31, (BD Biosciences). Secondary antibodies were conjugated to Alexa Fluor 594 Streptavidin, Alexa Fluor 647 Streptavidin, Alexa Fluor 647 donkey anti-rat, and Alexa Fluor 488 donkey anti-rabbit (Jackson ImmunoResearch). For the quantification of GFAP, Glut-1 and IBA-1 immunoreactive areas, 3 fields (650 μm × 450 μm) within peri-infarct cortex were precisely taken from 3 independent tangential sections of each animal using a 40× objective with confocal microscopy (Nikon C2). The parameters for scanning were kept constant across treatment conditions. Single images were analyzed using ImageJ by investigators blind to all treatment groups. Average percentage of the fluorescent staining area per image was calculated for each condition.

### ELISA

Tissue was collected 3 weeks post striatal stroke from tissue ipsilateral and contralateral the stroke site from stroke plus vehicle, stroke plus PDE10A (1.0 mg/kg and 3.0 mg/kg), and control groups. Striatal tissue was dissected in a 1-mm radius and flash frozen in liquid nitrogen. Equal volumes of tissue were homogenized in 100 mL of homogenization buffer (Complete Protease Inhibitor Tablet [Invitrogen]) dissolved in radio immunoprecipitation assay (RIPA) buffer (Sigma) and incubated on ice for 30 minutes, followed by a 5-minute spin at 14,000 rpm. The supernatant was collected and total protein concentrations were determined using the Pierce™ BCA Protein Assay kit (ThermoFisher Scientific). BDNF was measured using the BDNF (mouse) enzyme-linked immunosorbent assay (ELISA) kit (Abnova) according to the manufacturer’s instructions. BDNF levels were determined relative to a standard curve constructed from measures of kit-supplied BDNF protein standards (0–1000 pg of BDNF protein) that were assayed simultaneously with the experimental samples. BDNF levels were expressed as picograms of BDNF per mg of sample protein. To measure proBDNF, p75NTR, and pTrkB protein levels, the following kits were used: BDNF (mouse) ELISA kit (Abnova), proBDNF Rapid ELISA Kit (Biosensis), Mouse NGF R/TNFRSF16 DuoSet ELISA (R&D Systems), and PathScan® Phospho-TrkB (panTyr) Sandwich ELISA (Cell Signaling Technologies). Protein levels were assayed according to the manufacturer’s instructions provided with each kit. These results are presented in Supplementary Tables 2–4.

## Results

### PDE10A Inhibition Improves Motor Recovery After Striatal Stroke With No Improvement in Motor Recovery After Cortical Stroke

Mice were given a stroke in the striatum [24] or in the motor cortex [5, 4, 25, 19] in separate cohorts (Supplemental Figure 1A–C). Behavioral testing of forelimb motor function was performed to 9 weeks post stroke to measure motor control using exploratory gait in forelimb function.

**Figure 1.**
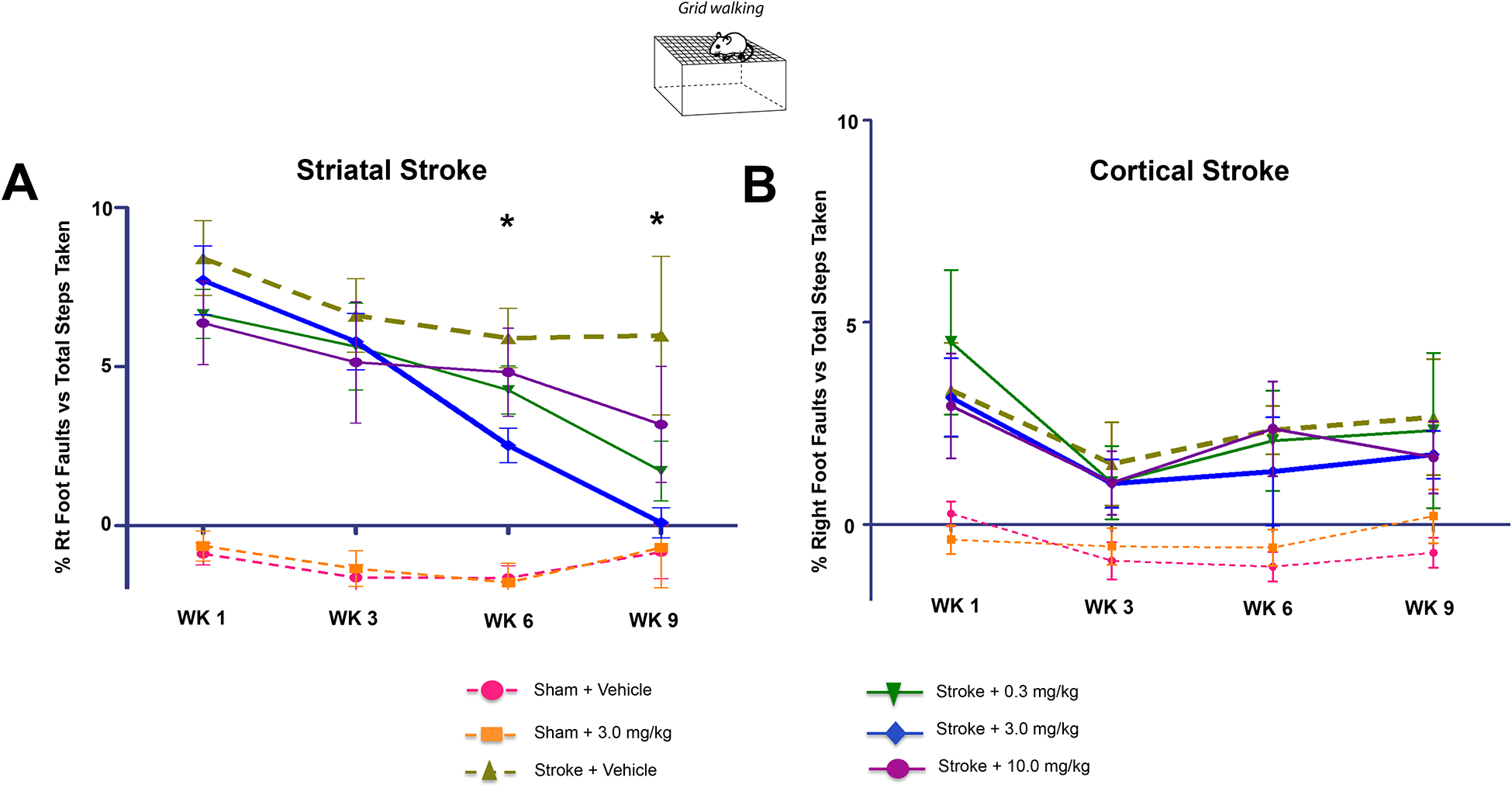
Functional effect of TAK-063 on forelimb use and gait post-striatal and cortical stroke. (A) In post-striatal stroke of the left hemisphere, the Grid Walking task revealed differences in gait observed for Stroke + Vehicle versus Stroke + 3.0 mg/kg at 6 weeks; *P = 0.04317 and at 9 weeks; *P = 0.03387 in post-striatal stroke. For overall differences between Stroke + Vehicle versus Stroke + 3.0 mg/kg, *P* = 0.0129. (B) In post-cortical stroke of the left hemisphere, the Grid Walking task revealed no differences in Stroke + Vehicle versus treatment groups. Error bars represent mean ± SEM for n = 8–10 per group in striatal stroke model and n = 8–11 in cortical stroke model. Data are reported as difference from baseline. Data were analyzed by GLMs with Tukey’s HSD. In (A) z = 2.901 at 6 weeks, z = 2.984 at 9 weeks

A competitive inhibitor of PDE10A, TAK-063 [16, 17], was administered i.p. beginning 5 days after stroke, a time point at which brain sensitivity to damage-enhancing effects of plasticity drugs is lost [4]. One week after surgery, the *Grid-Walking Task*, a measure of limb use in gait, showed contralateral forelimb deficits in stroke + vehicle, stroke + 0.3 mg/kg, stroke + 3.0 mg/kg, and stroke +10 mg/kg groups compared with sham + vehicle and sham + 3.0 mg/kg treatment controls (Figure 1A). In striatal stroke, a significant improvement was observed at the individual 6-week (P = 0.04317) and 9-week (P = 0.03387) time points in stroke + 3.0 mg/kg versus stroke + vehicle controls. TAK-063 showed significant improvement in overall recovery, with a difference in stroke + 3.0 mg/kg over the 9-week treatment compared with stroke + vehicle controls (Figure 1A, n = 8–10 for each treatment group, *P* = 0.0129). TAK-063 did not enhance recovery of motor function in cortical stroke (Figure 1B, n = 8–11, *P* = 0.9934). Overall, the results obtained from the *Grid-Walking Task* indicate that a daily dose of TAK-063 leads to improvement in gait and forelimb motor control in striatal stroke but not cortical stroke. A separate behavioral test, the *Cylinder Task*, a measure of forelimb use in exploratory rearing, was also performed for both striatal and cortical stroke mice. The Cylinder test showed great variability between animals in motor performance and across time points. The stroke + 3.0 mg/kg dose of TAK-063 produced a tendency toward recovery of function in the affected forelimb in the striatal stroke over the 9-week treatment compared with stroke + vehicle controls (Supplementary Figure 2A, *P* = 0.0558). No differences were observed for the stroke + 0.3 mg/kg and 10 mg/kg treatment groups in the striatal stroke model. There were no differences between the groups in the Cylinder test in cortical stroke mice (Supplementary Figure 2B).

### PDE10A Inhibition Leads to Increases in BDNF

**Figure 2.**
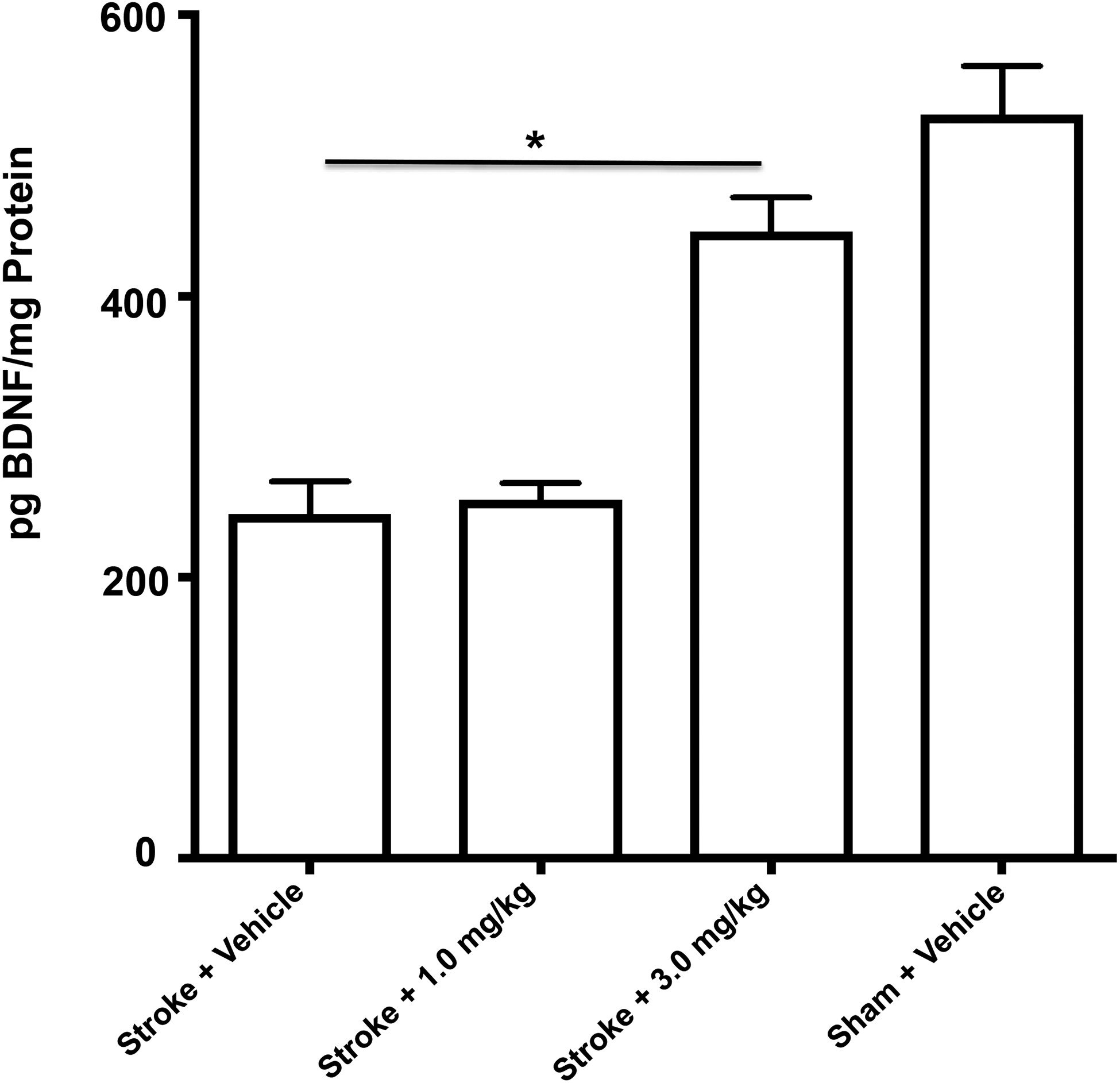
TAK-063 treatment-mediated alterations in BDNF expression in striatal tissue. BDNF expression levels measured with tissue ELISA showed elevation at 3 weeks post-striatal stroke in striatal tissue, ipsilateral to the infarct. Treatment with TAK-063 resulted in a significant increase in BDNF levels in the Stroke + 3.0 mg/kg versus Stroke + Vehicle group in ipsilateral striatum; *P* = 0.005, corrected for multiple comparisons (df=18, t value = 5.982). Error bars represent mean ± SEM for n = 10 per group.

Within striatum, PDE10A is predominantly expressed in medium spiny neurons, the major integrative and output source of the striatum [26, 27]. We next investigated if treatment with TAK-063 leads to increases in BDNF levels at 3 weeks following striatal stroke, a time point where heightened levels of BDNF post-stroke have been reported [6] and just before significant behavioral recovery with this drug is observed (Figure 1A; Supplemental Figure 3). There is a significant loss of BDNF levels in the ipsilateral striatum with stroke (Figure 2). Striatal stroke + 3.0 mg/kg of TAK-063 significantly induced BDNF in ipsilateral, but not contralateral striatum (Figure 2; Supplemental Figure 4). This low level of striatal BDNF does not change significantly with TAK-063 delivered at a dose that is ineffective in promoting behavioral recovery (i.e., at 1.0 mg/kg) (Figure 2).

### PDE10A Inhibition Alters Motor System Connections after Stroke

**Figure 3.**
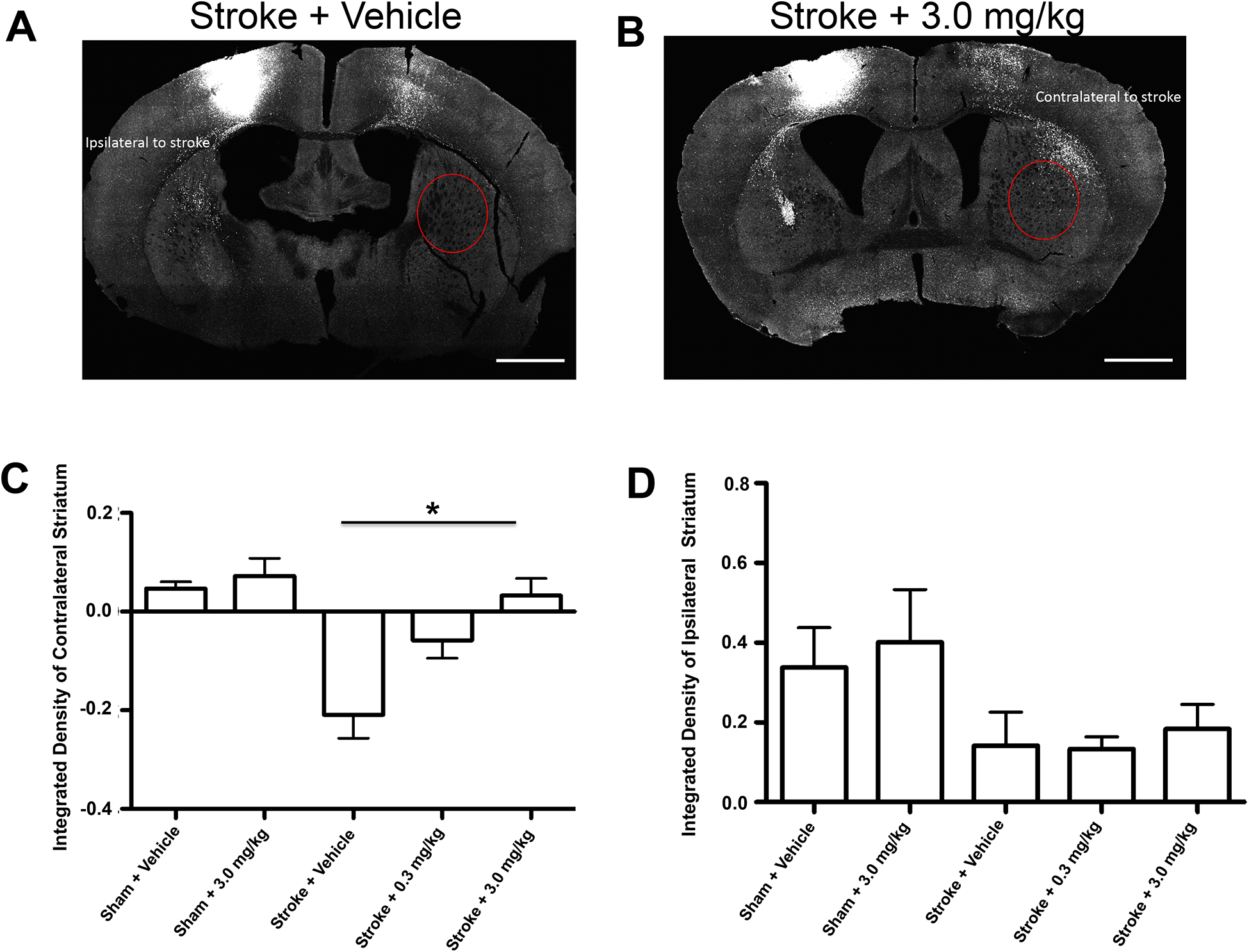
Motor connections post-striatal stroke. (A) Image of BDA-labeled connections from the motor cortex of Stroke + Vehicle and (B) Stroke + 3.0 mg/kg mice. Circles show area measured for axonal fibers in contralateral striatum. Data were normalized to integrated density of the BDA injection site for each treatment group. (C) Total BDA^+^ fibers were increased at 6 weeks post-striatal stroke in the contralateral cortical tissue in Stroke + 3.0 mg/kg versus Stroke + Vehicle; *P = 0.0009, F(4, 17) = 10.69. (D) Ipsilateral striatum showed no differences between groups F(4, 17) = 10.69. Error bars represent mean ± SEM for n = 4–5 per group. Scale bar = 50 μm and applies to all photomicrographs.

Neural repair after cortical stroke involves axonal sprouting within the ipsilateral peri-infarct cortex [19], and tissue reorganization in other rodent models of ischemic brain lesions also includes axonal sprouting in cortical projections to the contralateral striatum from the stroke site [28]. Increases in BDNF promote axonal sprouting after motor cortex and striatal lesions [29]. To determine if the improvement in forelimb motor recovery and induction of BDNF in the striatum following TAK-063 were associated with axonal sprouting after striatal stroke, the neuroanatomical tracer BDA was used to quantitatively map axonal projections from the forelimb motor cortex 6 weeks after stroke, a time of improved motor recovery (Figure 1A). The pattern of axonal sprouting in stroke and stroke +TAK-063 at the recovery-inducing dose of 3.0 mg/kg was compared with sham + vehicle and sham + 3.0 mg/kg TAK-063. Axonal connections were assessed in the density of BDA-positive axons projecting from primary motor cortex on the side of the stroke. The mean integrated density of labeled axons linearly correlates with axon number [30]. Data was normalized to the integrated density of the BDA injection site to account for differences in injection size (Figure 3A–D). Striatal stroke causes a loss in the projections from the motor cortex to the contralateral striatum (Figure 3C). Stroke + 3.0 mg/kg TAK-063 compared with stroke + vehicle showed significant increases (Figure 3C, n = 4–5 for each treatment group, *P* = 0.0009) in density of BDA positive fibers in the contralateral striatum (Figure 3C; data representative of two independent experiments). Axonal motor projections ipsilateral to stroke were mapped and no differences were observed in ipsilateral striatum (Figure 3D). These data suggest that TAK-063 either preserves the normal pattern of corticostriatal connections or that TAK-063 induces axonal sprouting in the partially damaged corticostriatal system and restores the absolute level of this system through compensatory axonal sprouting responses.

### PDE10A Inhibition Does Not Affect Astrocyte, Microglia, and Vasculature Patterns in the Striatum After Stroke

**Figure 4.**
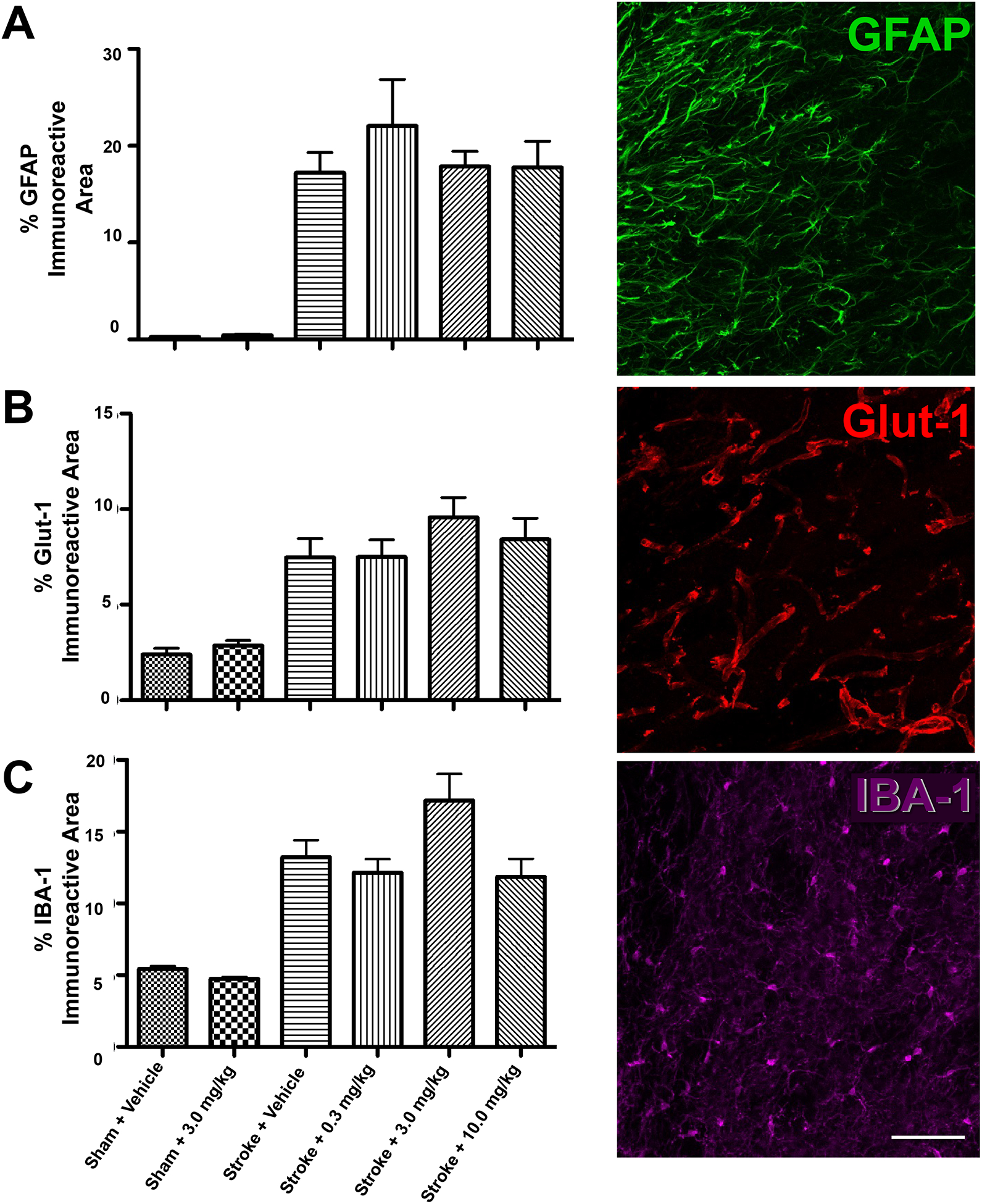
TAK-063 treatment does not alter mature astrocytes, microglia, or vessels in post-striatal stroke tissue. (A) Quantification of GFAP-positive astrocytes showed no differences between TAK-063 treatment groups in mice post-stroke (t value = 0.2513, df=11). In each panel, a representative tissue stain is shown to the right. (B) Glut-1 staining in peri-infarct tissue for quantification of vascular endothelial cells post-striatal stroke revealed no differences between treatment groups (t value = 1.461, df=11). (C) Quantification of IBA-1 immunoreactivity for microglia/macrophages showed no significant differences in microglia staining in peri-infarct striatal tissue (t value = 1.718, df=11). Error bars represent mean ± SEM for n = 8–10 per group. Images are taken from a representative stroke + vehicle condition. Scale bar = 50 μm and applies to all photomicrographs. Inset in top row is a schematic of coronal section with striatal stroke and box indicates location of tissue photomicrogaphs and measurements.

BDNF can promote astrocytosis, microglial activation, and vascular remodeling both after stroke and in normal brain tissue [6]. We measured TAK-063 effects in the brain immediately adjacent to the stroke site at 9 weeks post-stroke, a time-point of functional motor recovery. Stroke + 0.3 mg/kg, stroke + 3.0 mg/kg, and stroke + 10 mg/kg groups, when compared with stroke + vehicle, had no effect on patterns of vascular, microglial and astrocyte staining (Figure 4A–C). A similar lack of tissue effect in these measures was seen in the cortical stroke model (data not shown).

### Effects of PDE10A Inhibition Post-Striatal Stroke on Angiogenesis

**Figure 5.**
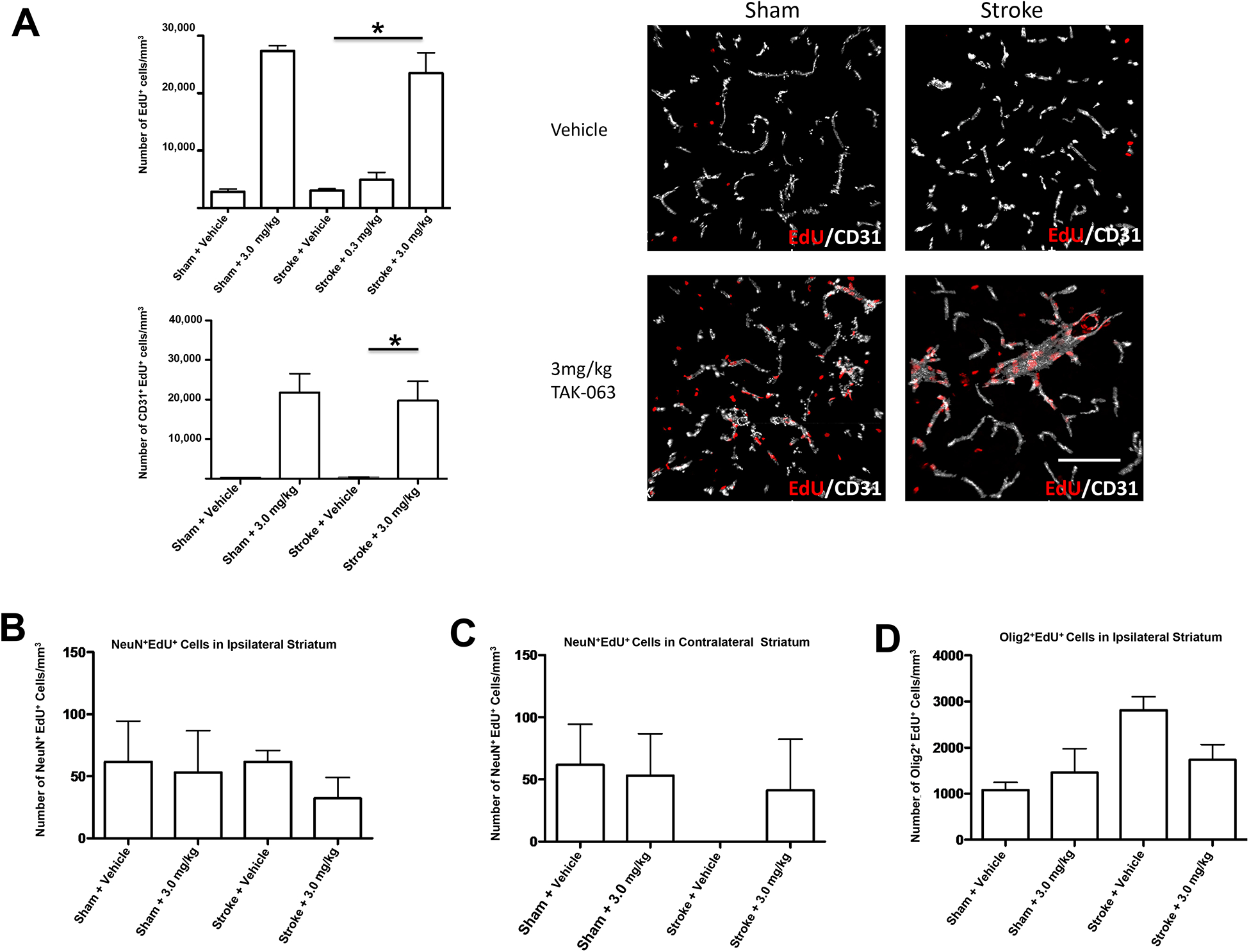
Effects of PDE10A inhibition on angiogenesis, neurogenesis, and gliogenesis in post-striatal stroke. (A, upper panel) Total EdU^+^ cells were increased at 6 weeks post-striatal stroke in contralateral striatum in Stroke + 3.0 mg/kg versus Stroke + Vehicle; *P < 0.0001 (F(4,15) = 32.6) (A, lower panel) EdU^+^CD31^+^ cells were increased at 6 weeks post-striatal stroke in striatal tissue contralateral to the infarct. Representative images of EdU^+^CD31^+^ cells in contralateral striatum. From top left: Sham + Vehicle, bottom left: Sham + 3.0 mg/kg. top right: Stroke + Vehicle, bottom right: Stroke + 3.0 mg/kg. (A, lower panel) Treatment with TAK-063 resulted in a significant increase in EdU^+^CD31^+^ cells in the Stroke + 3.0 mg/kg versus Stroke + Vehicle group; *P = 0.0201. No differences were observed in Sham + 3.0 mg/kg and Stroke + 3.0 mg/kg. Error bars represent mean ± SEM for n = 3–5 per group, F(3,9) = 9.700. (B, C) NeuN^+^ EdU^+^ cells showed no differences between groups at 6 weeks post-striatal stroke in contralateral and ipsilateral striatum. Error bars represent mean ± SEM for n = 3 per group, (B) F(3,8) = 0.3004, (C) F(3,8) = 0.7663. (D) Olig2^+^ EdU^+^ cells showed no differences between groups in ipsilateral striatum. Error bars represent mean ± SEM for n = 3–4 per group, F(3, 10) = 4.040. Scale bar = 50 μm and applies to all photomicrographs.

Angiogenesis plays a role in tissue reorganization in stroke and has been linked to recovery in associational studies in humans [31]. There was no change in vascular structure with TAK-063 treatment (Figure 4B). There may be an induction of proliferative angiogenesis (endothelial cell division) in the absence of changes in the overall morphology of the vascular bed. To determine endothelial proliferation in the tissue adjacent to the infarct, mice were given a striatal stroke and were treated with a marker of cell proliferation, EdU, for 6 weeks (Supplemental Figure 5A–C for overall study timeline and experimental groups). Stroke + 3.0 mg/kg TAK-063 increased the total number of proliferative cells in the contralateral striatum compared with stroke + vehicle and stroke + 0.3 mg/kg; there was no significant difference between stroke + vehicle and stroke + 0.3 mg/kg (Figure 5A, upper panel). Co-localization of the EdU nuclear signal with the endothelial maker CD31, showed a significant increase in proliferating endothelial cells with stroke + 3.0 mg/kg TAK-063 compared with stroke + vehicle (Figure 5A, lower and right panels). Interestingly, in the non-stroke (control) condition, TAK-063 induced angiogenesis in the striatum (Figure 5A, lower and right panels) (n = 3–5 per treatment group, *P* < 0.0001). No differences were observed in ipsilateral striatum after stroke.

### Effects of PDE10A Inhibition on Post-Striatal Stroke Neurogenesis and Gliogenesis

Angiogenesis is associated with neurogenesis after stroke [32], and during brain development, angiogenesis regulates oligodendrocyte precursor cell (OPC) differentiation and myelination [33]. With the angiogenesis induced by TAK-063 after stroke, we examined neurogenesis and OPC proliferation in the region of significant angiogenesis (the contralateral striatum). There were no differences in a maker of mature neurons, NeuN^+^ co-localized with EdU^+^ cells, in TAK-063-treated mice compared with controls in either striatal hemisphere (Figure 5B, C). Olig2 is a broad marker of cells throughout all but the last stages of OPC differentiation [34]. Quantification of Olig2^+/^EdU^+^ cells indicates that there is no significant effect of stroke or of TAK-063 treatment on OPC proliferation (Figure 5D).

### Delayed PDE10A Inhibition Does Not Affect Infarct Volume

**Figure 6.**
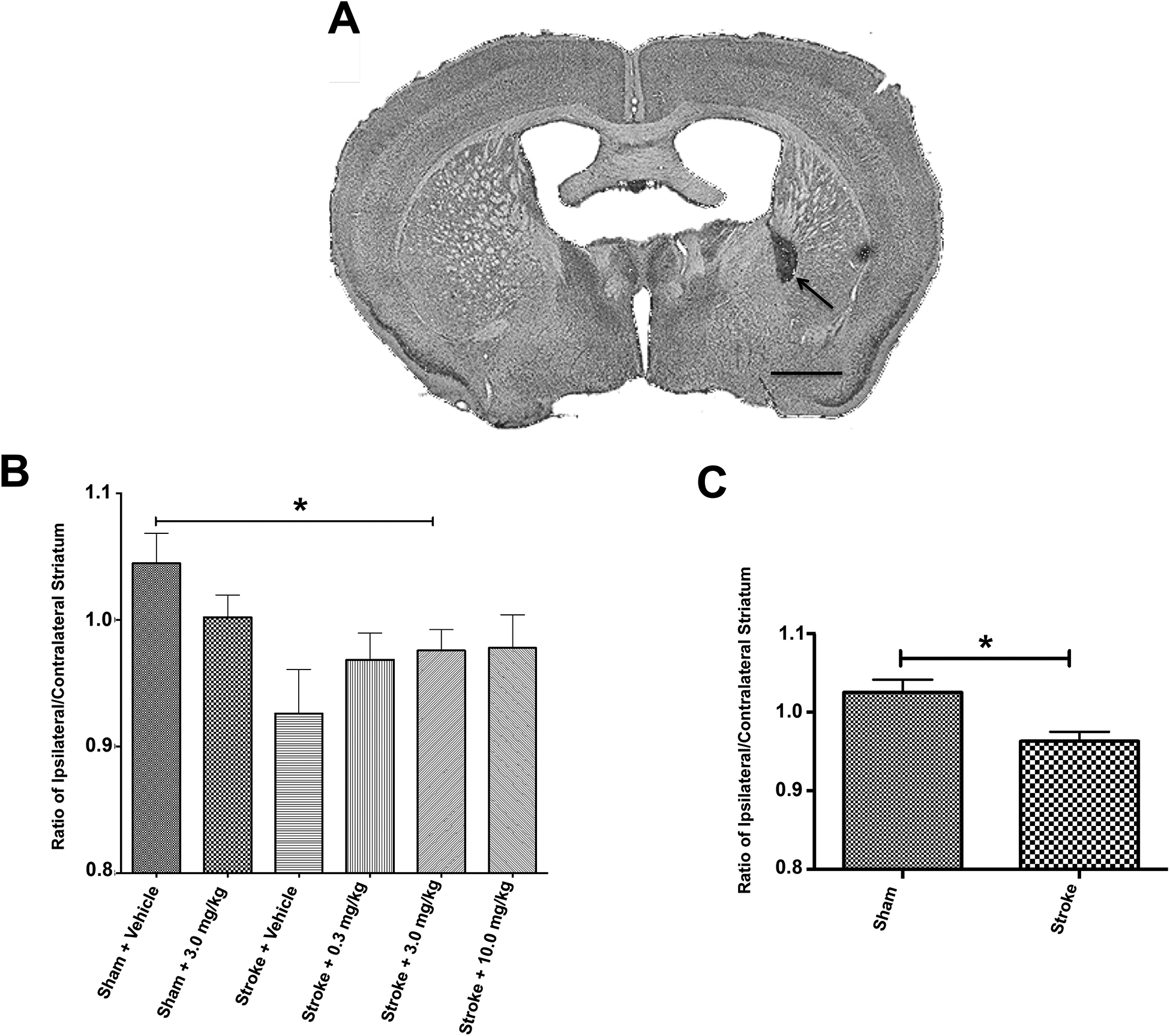
Degree of infarct size in TAK-063-treated mice. (A) Top panel shows Nissl-stained brain tissue section from representative stroke + 3.0 mg/kg group at 10 weeks post-stroke. The arrow indicates the core of the infarct. The lower panels quantifies the degree of stroke. The Y-axis indicates the normalized ratio of the ipsilateral stroked hemisphere over the contralateral non-stroked hemisphere. Data is shown are measurements at −0.22 mm to −0.82 mm from Bregma. Stroke leads to a loss of tissue volume in the hemisphere ipsilateral to the stroke. Lower values indicate loss of tissue volume. Data show that there was no effect between the treatment groups; however (B) Stroke + 3.0 mg/kg and Stroke + Vehicle group shows a significant difference compared to Sham + Vehicle; *P = 0. 0146; *P = 0.0053; n = 8–10 per group. (C) The combined stroke groups versus the combined sham control groups (n =18 and 32, *P = 0.001). All p-values corrected for multiple comparisons. Error bars represent mean ± SEM. Scale bar = 50 μm and applies to all photomicrographs.

Brain volume was assessed between TAK-063 treatment groups as a determination of stroke size for both the striatal and cortical stroke models at 9 weeks post-stroke. At this late stage after stroke, the size of the stroke and of any secondary tissue loss can be determined by comparing the ipsilateral cerebral striatum to the contralateral cerebral striatum. No differences were found in the area of infarct between the striatal stroke groups irrespective of treatment. However, a statistical significance was observed as expected in stroke vs. sham groups: in both the stroke + 3.0 mg/kg TAK-063 and stroke + vehicle compared with sham + vehicle controls (Figure 6A–C). Nissl-stained brain tissue from the cortical stroke model was also examined and similar results were obtained (Supplementary Figure 6).

## Discussion

Inhibition of PDE10A is site-specific in its role of improving recovery after stroke. The PDE10A inhibitor TAK-063 promotes recovery of motor function after striatal stroke but not cortical stroke. This improvement in motor function with PDE10a inhibition is associated with elevated levels of BDNF in the striatum that has the stroke, and in enhanced angiogenesis and axonal connections in the striatum contralateral to the stroke. This study is to our knowledge one of the first reports of a pharmacological therapy for stroke recovery that is brain region-specific. Stroke in the striatum or basal ganglia is very common [15], damage in the striatum impairs motor learning, and striatal stroke is associated with a poorer response to neurorehabilitation [35–37].

There are several possible mechanisms of action for PDE10A inhibition in striatal stroke. There is not an effect of TAK-063 on infarct size, indicating the mechanism of action is not through an effect on cell death or damage in stroke. TAK-063 selectively enhances cAMP and cGMP signaling in medium spiny neurons of the striatum and enhances cognitive function, including declarative and working memory, attention, and measures of executive function [38]. In other models of neuronal degeneration, such as the severe R6/2 Huntington mouse, a relatively less specific PDE10A inhibitor improves memory function [39]. TAK-063 reduces neurodegeneration in the striatum and induces BDNF in the R6/2 mouse [40]. In striatal stroke, TAK-063 induces BDNF levels in the injured striatum, such that they are boosted from the low post-stroke state to that seen in control, non-injured striatum.

Stroke in the striatum leads to either no change in striatal BDNF levels [6] or to decreases in striatal BDNF (present data). This difference in striatal BDNF protein levels with striatal stroke may be due to differences in sampling the cortical projections to the striatum in the tissue blocks that are taken for BDNF measurement. In [6], we took tissue blocks from the striatum medial to the stroke, because the goal was to measure diffusion of BDNF from the hydrogel/BDNF biomaterial that was tested in that paper. There are few projections from sensorimotor cortex, or cortex in general, to this medial striatal area [38]. In the present study, we took tissue blocks for the BDNF measurement lateral to the stroke, because the goal was to determine if PDE10a inhibition activated BDNF in sensorimotor projections to the striatum. These projections terminate predominantly in dorsolateral striatum [38]. This difference in sampling site likely accounts for the differences in BDNF measurements in the same striatal stroke model across these two papers.

The effect of PDE10a inhibition on BDNF induction only in the stroke striatum, and not the contralateral side in the present data, likely is due to the damage from the stroke. BDNF is largely present in the striatum in cortical projections to this structure [39,40]. The activity and structural integrity of striatal neurons influences BDNF levels in cortex and in the cortical projections to the striatum—striatal damage or inactivity reduces BDNF levels, which are present in the cortical afferents [41]. PDE10a inhibition increases medium spiny neuron activity [42,43]. These data suggest that stroke in the striatum reduces BDNF expression in cortical neurons that project to the striatum, and that by increasing the activity of striatal neurons, PDE10a inhibition induces cortical BDNF levels or transport of this protein to the striatum.

BDNF is strongly associated with improved recovery in stroke as observed in many different studies and following systemic or intraparenchymal delivery [24]. A TAK-063 improvement in recovery after stroke may occur through the induction of BDNF in the partially damaged striatum. Increases in axonal connections and angiogenesis provide possible cellular mechanisms for enhanced recovery with PDE10A inhibition.

TAK-063 either prevents secondary loss of corticostriatal projections or enhances sprouting of preserved axons from the motor cortex ipsilateral to the striatal stroke into the contralateral striatum. These effects mean that the distributed connections of the motor cortex are closer to the control, non-stroke brain in mice with striatal stroke that are treated with the dose of TAK-063 that promotes recovery. However, there was no effect of this drug on motor system connections to the striatum with the stroke, and this might be expected to be more directly correlated with improved motor control. A similar contralateral effect was seen in angiogenesis: TAK-063 treatment enhanced angiogenesis in the striatum contralateral to the stroke but not in peri-infarct striatum.

In unilateral striatal damage, such as in Parkinson’s disease models, the contralateral striatum responds with an induction in plasticity associated genes [47]. Motor projections to contralateral striatum may be associated with bimanual motor control of the limbs and integration of somatosensory and motor inputs [48]. Axonal sprouting in this crossed or contralateral corticostriatal system is seen in cortical ischemic lesions [28, 49]. It may be that enhanced motor system connectivity to the contralateral striatum or changes in synaptic plasticity are an important element in motor recovery in this striatal stroke.

Brain PDEs catalyze the hydrolysis of cAMP and cGMP. These two pathways are associated with induction of genes that enhance synaptic plasticity, such as the transcription factor CREB, BDNF, and through modulation of nitric oxide signaling [50]. Eleven families of mammalian PDEs are encoded by 21 genes that may be modified to form an estimated 100 different protein products [50]. PDE2A and PDE4 are localized within forebrain regions of the cortex and the hippocampus. PDE4 is also found in the brainstem, which accounts for vomiting, the major side effect of PDE4 inhibition. A PDE4 inhibitor, rolipram, has been used in stroke models and may reduce early stroke damage and promote tissue reorganization [51]. PDE10A inhibitors are being developed for psychiatric disease and are considered as possible therapeutics in Huntington’s disease [50, 46].

The application of a PDE10A inhibitor in the subacute phase of stroke, after cell death and secondary injury have taken place, provides an opportunity for selective manipulation of neuronal signaling systems associated with synaptic plasticity particularly strong in the basal ganglia or striatum. The present findings suggest the possibility of a neural repair or recovery therapy in stroke that is distinct based on stroke subtype, with subcortical-basal ganglionic stroke receiving a different drug than cortical or possibly white matter stroke [52].

## Supplementary Material

12975_2020_819_MOESM1_ESM
