# Correction to: Phosphodiesterase 10A Inhibition Leads to Brain Region-Specific Recovery Based on Stroke Type

**DOI:** 10.1007/s12975-020-00858-1

**Published:** 2020-10-29

**Authors:** Shirin Z. Birjandi, Nora Abduljawad, Shyama Nair, Morteza Dehghani, Kazunori Suzuki, Haruhide Kimura, S. Thomas Carmichael

**Affiliations:** 1 Departments of Neurology and of Neurobiology, David Geffen, School of Medicine at UCLA, Los Angeles, CA USA; 2 Departments of Psychology and of Computer Science, University of Southern California, Los Angeles, CA USA; 3 Neuroscience Drug Discovery Unit, Research, Takeda Pharmaceutical Company Limited, Fujisawa, Kanagawa Japan

**Correction to: Transl. Stroke Res**

10.1007/s12975-020-00819-8

The article “Phosphodiesterase 10A Inhibition Leads to Brain Region-Specific Recovery Based on Stroke Type”, written by Shirin Z. Birjandi, Nora Abduljawad, Shyama Nair, Morteza Dehghani, Kazunori Suzuki, Haruhide Kimura and S. Thomas Carmichael, was originally published electronically on the publisher’s internet portal on 6 May 2020 without open access. With the author(s)’ decision to opt for Open Choice the copyright of the article changed on 24 September 2020 to © The Author(s) 2020 and the article isforthwith distributed under a Creative Commons Attribution 4.0 International License, which permits use, sharing, adaptation, distribution and reproduction in any medium or format, as long as you give appropriate credit to the original author(s) and the source, provide a link to the Creative Commons licence, and indicate if changes were made. The images or other third party material in this article are included in the article’s Creative Commons licence, unless indicated otherwise in a credit line to the material. If material is not included in thearticle’s Creative Commons licence and your intended use is not permitted by statutory regulation or exceeds the permitted use, you will need to obtain permission directly from the copyright holder. To view a copy of this licence, visit http://creativecommons.org/licenses/by/4.0.

The original article has been corrected.

Open Access This article is licensed under a Creative Commons. Attribution 4.0 International License, which permits use, sharing, adaptation,distribution and reproduction in any medium or format, as long as you give appropriate credit to the original author(s) and the source, provide a link to the Creative Commons licence, and indicate if changes were made. The images or other third party material in this article are included in the article's Creative Commons licence, unless indicated otherwise in a credit line to the material. If material is not included in the article's. Creative Commons licence and your intended use is not permitted by statutory regulation or exceeds the permitted use, you will need to obtain permission directly from the copyright holder. To view a copy of this licence, visit http://creativecommons.org/licenses/by/4.0.

