# Supplementary material for: Phosphodiesterase 10A Inhibition Leads to Brain Region-Specific Recovery Based on Stroke Type
Source: Transl Stroke Res. 2020 May 6;12(2):303–15. doi: 10.1007/s12975-020-00819-8 (PMC7644574; doi:10.1007/s12975-020-00819-8)
Supplement: Supplementary file 1 — (PDF 3122 kb) [file 12975_2020_819_MOESM1_ESM.pdf]

Birjandi et al Supplement.
Supplemental Figures 1 – 7
Tales 1-4

Supplemental Figure Captions

Figure 1. Timeline of Striatal and Cortical Strokes. (A) Experimental Groups for TAK-063 treatment for both striatal and cortical strokes. (B) Overall study timeline for TAK-063 treatment post-striatal and (C) cortical stroke. Baseline behavior testing was assessed 1 week (Wk) prior stroke. Once daily i.p. TAK-063 treatment began 5 days post-stroke for 9 Wks. Behavior testing was performed on Wks 1, 3, 6, and 9. BDA injections were performed upon completion of behavior testing and mice were euthanized at 10 WKs post stroke.

Figure 2. Cylinder Task in Behavioral Recovery. Overall this task showed great variability between animals and across time points. (A) In post-striatal stroke of the left hemisphere, the Cylinder task revealed that Stroke + 3.0 mg/kg versus Stroke + Vehicle had an overall increased right paw use over the 9-week treatment; P = , $P = 0.0558$. (B) In post-cortical stroke of the left hemisphere, the Cylinder task revealed no differences in Stroke + Vehicle versus treatment groups. Data were analyzed by GLMs with Tukey's HSD.

Figure 3. Timeline of Striatal BDNF Quantification. (A) Experimental Groups for TAK-063 treatment for striatal strokes and overall study timeline for BDNF quantification in TAK-063 treatment post-striatal stroke. Once daily i.p. TAK-063 treatment began 5 days post-stroke for 3 weeks (Wks). Behavior testing was performed on Wks 1, 3, 6, and 9. Mice were euthanized at 3 Wks post stroke.

Figure 4. BDNF Levels in Contralateral Striatum. Treatment with TAK-063 resulted no differences in BDNF levels in Stroke + Vehicle compared to other treatment groups in contralateral striatum. Error bars represent mean ± SEM for n = 3-4 per group.

Figure 5. Timeline of Angiogenesis, Neurogenesis, and Gliosis Study. (A) Experimental Groups for EdU tracking for TAK-063 treatment post-striatal stroke. (B) Overall study timeline for Neurogenesis studies of TAK-063 (PDE10Ai-01) treatment post-striatal stroke. Striatal strokes were performed day 0 and starting day 2, mice were given twice daily EdU i.p. for 5 consecutive days. Starting day 7 post-striatal stroke, EdU was added in to drinking water and changed every 48 hrs. Once daily i.p. TAK-063 treatment began 5 days post-stroke for 6 weeks (Wks). Mice were euthanized at 6 Wks post stroke.

Figure 6. Cortical Stroke Infarct Volumes. (A) The graph quantifies the degree of stroke. The Y-axis indicates the normalized ratio of the ipsilateral stroked hemisphere over the contralateral non-stroked hemisphere. Data show that there was no effect between the treatment groups; however (A) Stroke + 3.0 mg/kg and Stroke + Vehicle group shows a significant difference compared to Sham + Vehicle measured from 1.10 mm to -2.06 mm

from Bregma; *P = 0.0003; *P = 0.0001; n = 10-11 per group. (B) The combined sham controls versus the combined stroke groups (n = 20 and 42, *P = 0.0001). All p-values corrected for multiple comparisons. Error bars represent mean ± SEM.

Figure 7. Striatal Stroke Infarct Volumes. The graph quantifies the degree of stroke. The Y-axis indicates the normalized ratio of the ipsilateral stroked hemisphere over the contralateral non-stroked hemisphere. Data show that there was a significant effect between the Sham + Vehicle compared to Stroke + Vehicle measured from -0.22 mm to -1.94 from Bregma; *P = 0.0036; n = 8-10 per group.

Table 1. Statistical tests performed with corresponding t-values, df, F, and z values where appropriate.

Tables 2-4. Raw data for ELISA studies of proBDNF, p75NTR and pTrkB. None of this data shows statistical significance across experimental groups.

Details of the animals excluded from the study:

The experimental groups and data behind Table 1 are:
Figures 1(A), 4, and 6 are data from the same cohort of experiments. We started with an **n** of 10 for each group in the striatal studies. Post-stroke surgical complications (e.g. cut of vagus nerve, overdose of anesthesia, bleeding, etc) resulted in the following reductions of n's in each group:

Sham + Vehicle: n = 10
Sham + 3.0 mg/kg: n = 10
Stroke + Vehicle: n = 8
Stroke + 0.3 mg/kg: n = 9
Stroke + 3.0 mg/kg: n = 10
Stroke + 10 mg/kg: n = 9

Figures 1(B) and Supplemental 5: Note n's were increased (10 or 11) to cover possible initial surgical losses. Loss due to complications of surgery occurred in the Sham + 3.0 mg/kg (1 animal). Stroke + 0.3 mg/kg (2 animals) showed no evidence of stroke post mortem.

Sham + Vehicle: n = 10
Sham + 3.0 mg/kg: n = 9
Stroke + Vehicle: n = 10
Stroke + 0.3 mg/kg: n = 8
Stroke + 3.0 mg/kg: n = 11
Stroke + 10 mg/kg: n = 11

Figure 2 is representative of a separate cohort of experiments to determine BDNF protein levels. Fresh brains are needed for the ELISA assay, therefore a separate experimental

group needed to be run. Given that we saw significant differences in the Stroke + 3.0 mg/kg TAK-063 group in the behavioral data only, we repeated the study excluding the 0.3 mg/kg and 10 mg/kg groups since no functional recovery was observed in these mice. The 1.0 mg/kg group was added to demonstrate a dose dependent response. The n's remained 10 per group as no animals were lost due to post-stroke surgical complications.

Sham + Vehicle: n = 10
Sham + 3.0 mg/kg: n = 10
Stroke + Vehicle: n = 10
Stroke + 1.0 mg/kg: n = 10
Stroke + 3.0 mg/kg: n = 10

Supplemental Figure 3: is representative of a separate cohort of experiments to determine BDNF levels in the contralateral hemisphere of striatal stroked mice given daily TAK-063. The sample size initially was 4. In the Stroke + 1.0 mg/kg 1 mouse was removed from the study as no striatal stroke was observed post mortem.

Sham + Vehicle: n = 4
Sham + 3.0 mg/kg: n = 4
Stroke + Vehicle: n = 4
Stroke + 1.0 mg/kg: n = 3
Stroke + 3.0 mg/kg: n = 4

Figure 3 is representative of a separate cohort of experiments to determine axonal sprouting via BDA. The sample size initially was 5. This separate cohort was repeated, to confirm the results obtained from the first cohort of BDA studies as the n's were quite low. The below loss of n's in the groups were due to no presence of BDA, therefore quantification of axonal sprouting could not be performed on those mice.

Sham + Vehicle: n = 5
Sham + 3.0 mg/kg: n = 4
Stroke + Vehicle: n = 4
Stroke + 0.3 mg/kg: n = 4
Stroke + 3.0 mg/kg: n = 5

Figure 5 is representative of a separate cohort of experiments to determine neurogenesis, gliogenesis, and angiogenesis. In order to determine cell proliferation, animals needed to be administered EdU daily and could not be paired with the initial studies done in Figure 1, 4, and 6. The sample size initially was 5 for each group. During stroke the normal n's were lost due to surgical complications resulting in the below n's. Additionally, 1 animal from the Stroke + Vehicle and 2 animals from the Stroke + 0.3 mg/kg were found to have no evidence of stroke post mortem and were excluded from the study.

Sham + Vehicle: n = 4
Sham + 3.0 mg/kg: n = 4

Stroke + Vehicle: n = 4
Stroke + 0.3 mg/kg: n = 3
Stroke + 3.0 mg/kg: n = 5

Supplemental Figure 1

A

Experimental Groups For Striatal and Cortical Strokes

Stroked mice divided into the following groups:

- Group 1: Sham + Vehicle*
- Group 2: Sham + 3.0 mg/kg
- Group 3: Stroke + Vehicle
- Group 4: Stroke + 10.0 mg/kg
- Group 5: Stroke + 3.0 mg/kg
- Group 6: Stroke + 0.3 mg/kg

B

Overview of Experimental Design (Striatal Strokes)

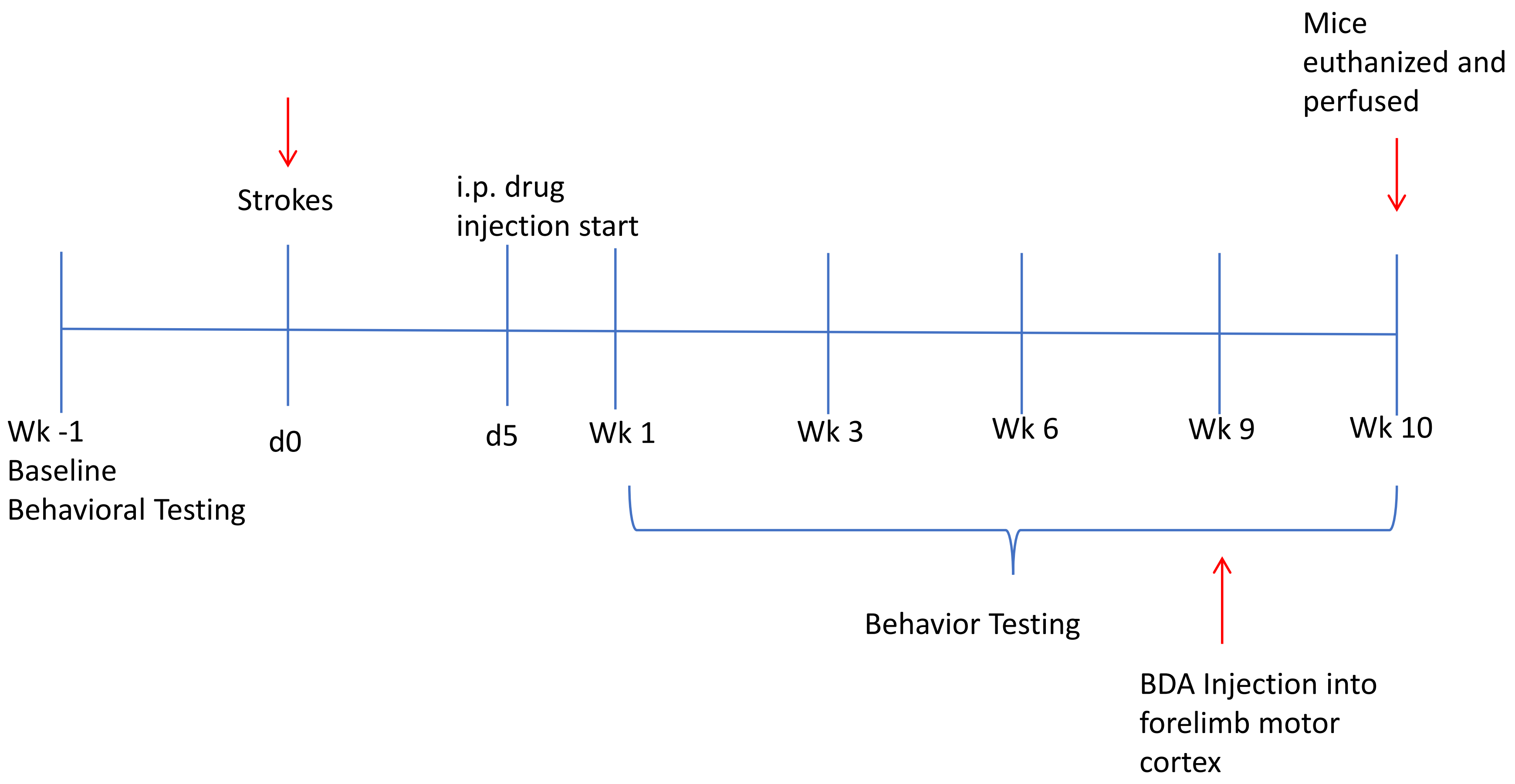

C

Overview of Experimental Design (Cortical Strokes)

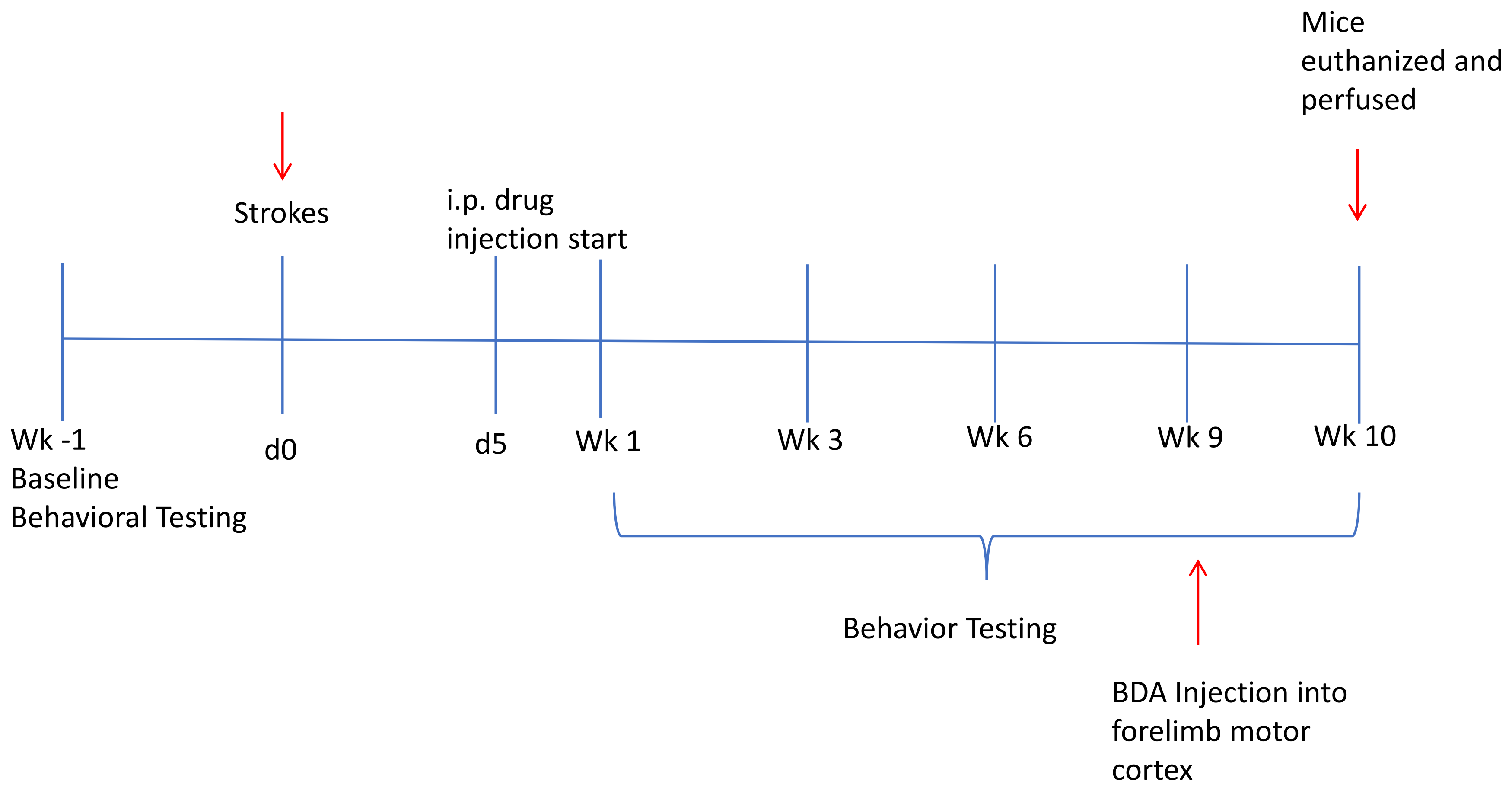

Supplemental Figure 2

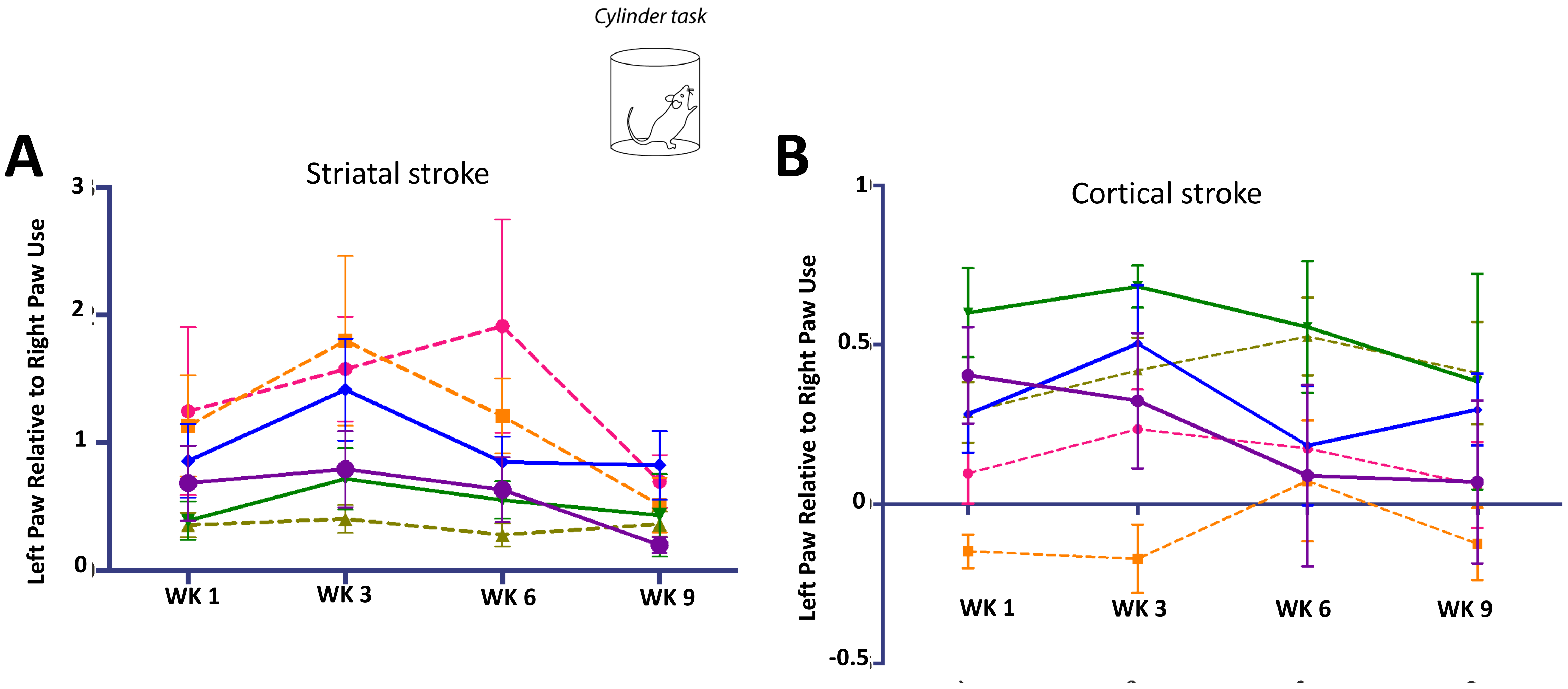

Supplemental Figure 3

Quantification of BDNF (Striatal Strokes)

A

Stroked mice were divided into the following groups:

<u>Group 1:</u> Sham + Vehicle

<u>Group 2:</u> Stroke + Vehicle

<u>Group 3:</u> Stroke + 1mg/kg

<u>Group 4:</u> Stroke + 3mg/kg

B

Experimental Design

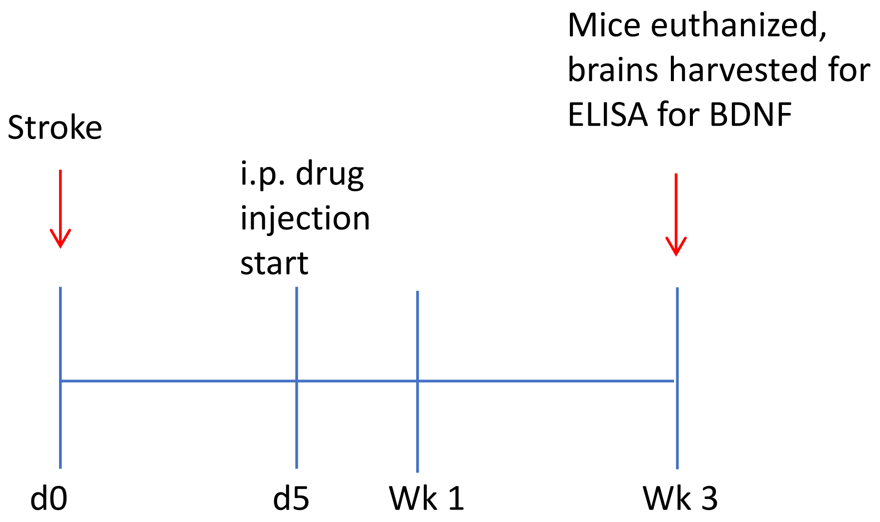

Supplemental Figure 4

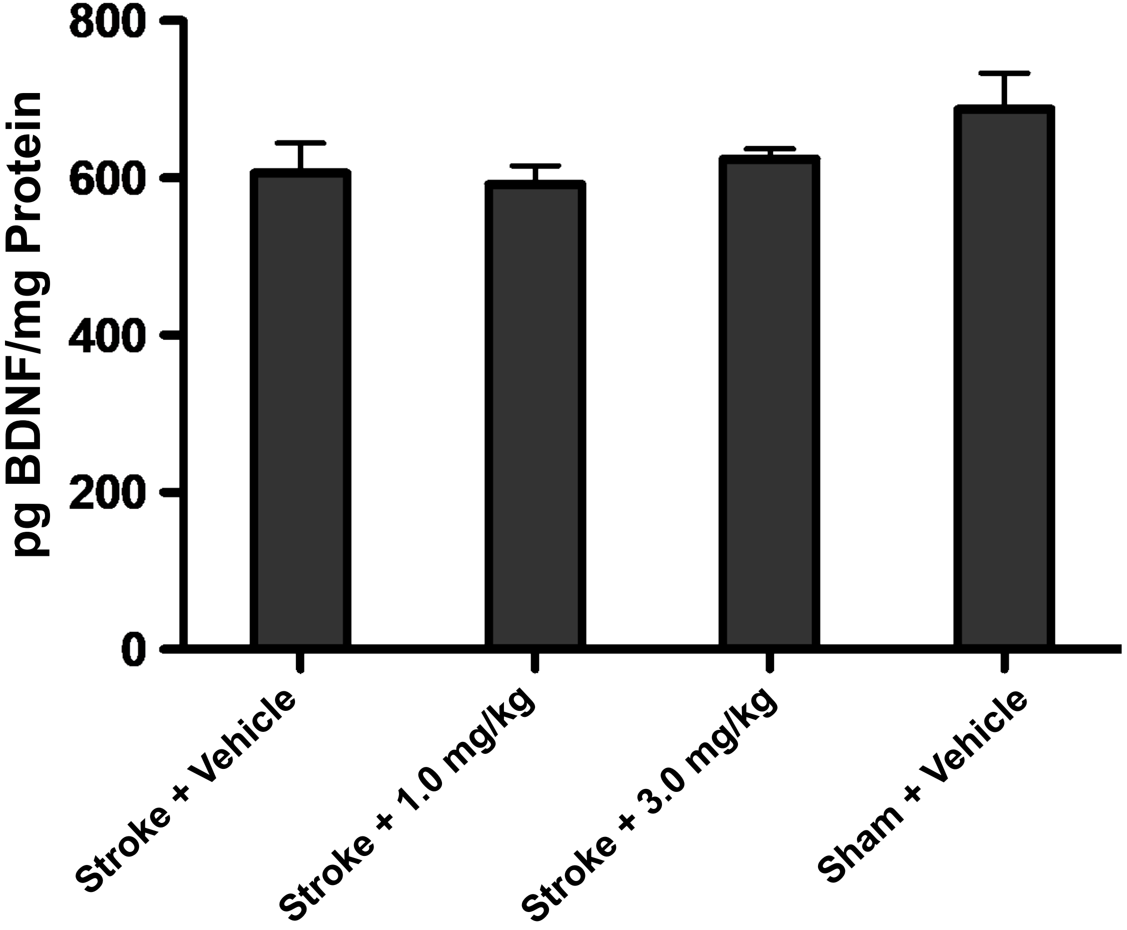

Supplemental Figure 5

A

Experimental Groups
(Neurogenesis – Striatal Strokes)

Stroked mice divided into the following groups:

- Group 1: Sham + Vehicle*
- Group 2: Sham + 3.0 mg/kg
- Group 3: Stroke + Vehicle
- Group 4: Stroke + 3.0 mg/kg
- Group 5: Stroke + 0.3 mg/kg

Overview of Experimental Design
(Angiogenesis, Neurogenesis, and Gliosis – Striatal Strokes)

B

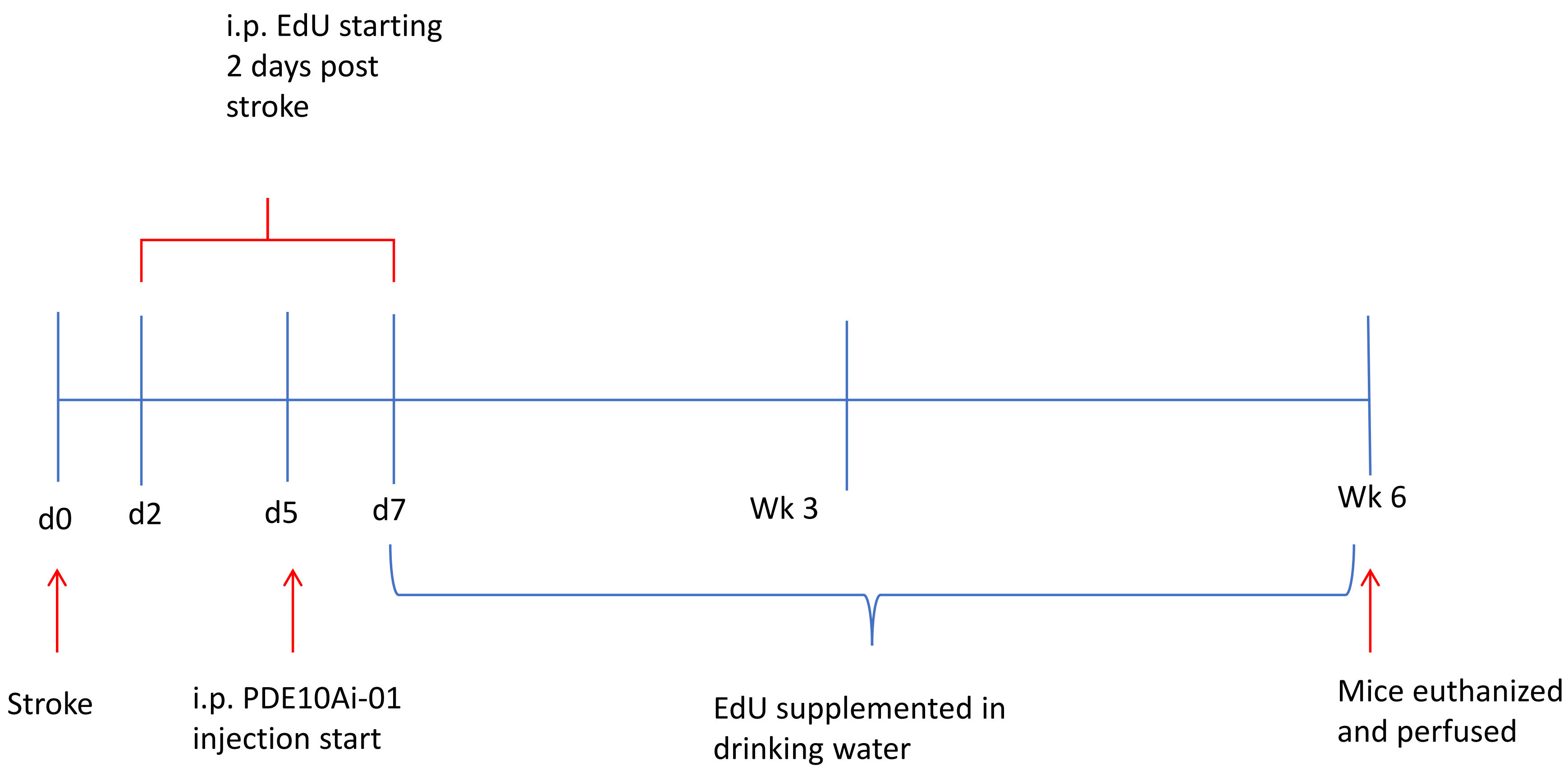

Supplemental Figure 6

Cortical Stroke Infarct Volumes

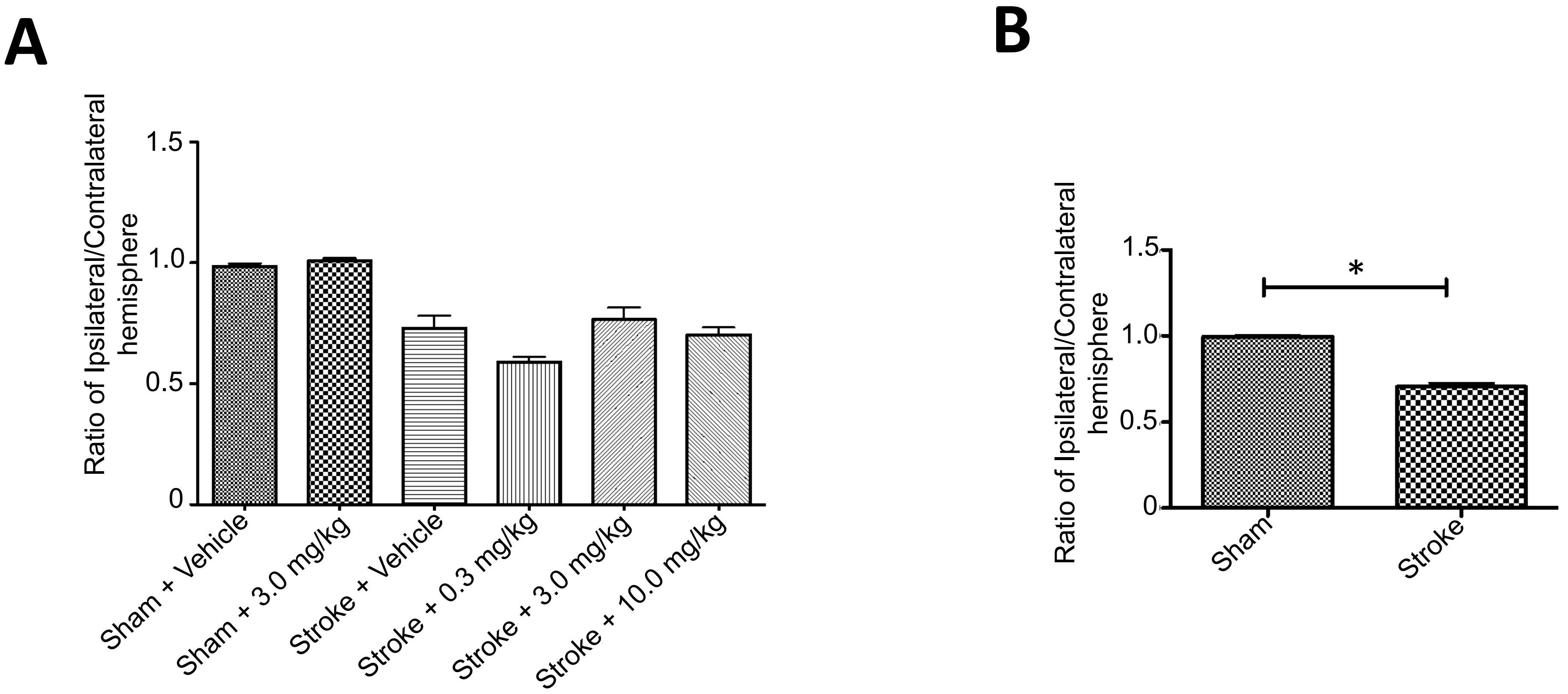

Supplemental Figure 7

Striatal Stroke Infarct Volumes (-0.22 mm to -1.94 mm)

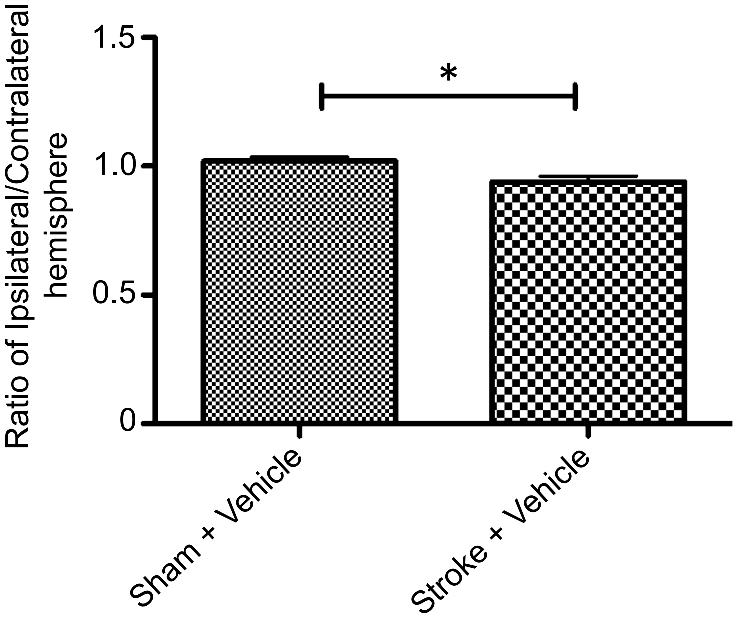

Table 1

Figure	Statistical Test	t Value	df Value	F Value	z Value
Figure 1A	GLM	-	-	-	z = 2.901 at 6 weeks<br>z = 2.984 at 9 weeks
Figure 2	Unpaired t test	5.982	18	-	
Figure 3C	ANOVA	-	-	F(4, 17) = 10.69	
Figure 3D	ANOVA	-	-	F(4, 17) = 10.69	
Figure 4A	Unpaired t test	0.2513	11	-	
Figure 4B	Unpaired t test	1.461	11	-	
Figure 4C	Unpaired t test	1.718	11	-	
Figure 5A (top)	ANOVA	-	-	F(4,15) = 32.6	
Figure 5A (bottom)	ANOVA	-	-	F(3,9) = 9.700	
Figure 5B	ANOVA	-	-	F(3,8) = 0.3004	
Figure 5C	ANOVA	-	-	F(3,8) = 0.7663	
Figure 5D	ANOVA	-	-	F(3, 10) = 4.040	
Figure 6C	Unpaired t test	2.919	15	-	
Figure 6D	Unpaired t test	3.120	48	-	
S Figure 2	GLM	-	-	F(3, 11) = 1.511	
S Figure 6A (Sham vs. Stroke + 3.0 mg/kg)	Unpaired t test	4.118	19	-	
S Figure 6b (Sham vs. Stroke + Veh)	Unpaired t test	4.553	19	-	
S Figure 7	Unpaired t test	8.842	60	-	

Table 2

proBDNF levels by ELISA

Sample	AdjConc (pg/mL)	Tot. Protein (mg/mL)	proBDNF pg per mg total protein
Stroke + Vehicle Ipsi #1	100.233	1.667	**60.12777445**
Stroke + Vehicle Ipsi #2	50.664	2.038	**24.85966634**
Stroke + Vehicle Ipsi #3	56.036	1.94	**28.88453608**
Stroke + Vehicle Ipsi #4	93.006	2.815	**33.03943162**
Stroke + 1mg/kg Ipsi #1	136.551	1.848	**73.89123377**
Stroke + 1mg/kg Ipsi #2	73.308	1.806	**40.59136213**
Stroke + 1mg/kg Ipsi #3	185.352	2.382	**77.81360202**
Stroke + 1mg/kg Ipsi #4	86.161	2.223	**38.75888439**
Stroke + 3mg/kg Ipsi #1	75.5	2.135	**35.36299766**
Stroke + 3mg/kg Ipsi #2	58.541	1.93	**30.33212435**
Stroke + 3mg/kg Ipsi #3	49.833	1.797	**27.7312187**
Stroke + 3mg/kg Ipsi #5	36.645	2.074	**17.66875603**
Stroke + Vehicle Contra #1	68.654	1.995	**34.41303258**
Stroke + Vehicle Contra #2	71.518	2.259	**31.65914121**
Stroke + Vehicle Contra #3	116.092	2.424	**47.89273927**
Stroke + Vehicle Contra #4	53.724	2.125	**25.28188235**
Stroke + 1mg/kg Contra #1	68.43	2.543	**26.90916241**
Stroke + 1mg/kg Contra #2	100.546	2.133	**47.13830286**
Stroke + 1mg/kg Contra #3	58.233	2.458	**23.69121237**
Stroke + 1mg/kg Contra #4	52.665	2.289	**23.0078637**
Stroke + 3mg/kg Contra #1	75.028	2.169	**34.59105579**
Stroke + 3mg/kg Contra #2	48.571	2.285	**21.25645514**
Stroke + 3mg/kg Contra #3	50.167	2.281	**21.99342394**
Stroke + 3mg/kg Contra #4	48.443	2.233	**21.69413345**

Table 3

p75NTR levels by ELISA

Sample	AdjConc (pg/mL)	Tot. Protein (mg/mL)	p75NTR pg per mg total protein
Stroke + Vehicle Ipsi #1	1280.234	1.667	767.9868026
Stroke + Vehicle Ipsi #2	1316.615	2.038	646.0328754
Stroke + Vehicle Ipsi #3	1554.233	1.94	801.1510309
Stroke + Vehicle Ipsi #4	1633.752	2.815	580.3737123
Stroke + 1mg/kg Ipsi #1	1343.479	1.848	726.9908009
Stroke + 1mg/kg Ipsi #2	1130.493	1.806	625.9651163
Stroke + 1mg/kg Ipsi #3	1518.558	2.382	637.5138539
Stroke + 1mg/kg Ipsi #4	1727.118	2.223	776.9311741
Stroke + 3mg/kg Ipsi #1	1385.603	2.135	648.9943794
Stroke + 3mg/kg Ipsi #2	1447.199	1.93	749.8440415
Stroke + 3mg/kg Ipsi #3	972.927	1.797	541.4173623
Stroke + 3mg/kg Ipsi #6	1081.337	1.814	596.1063947
Sham + Vehicle Ipsi #1	1075.575	1.586	678.168348
Sham + Vehicle Ipsi #2	1249.055	1.915	652.2480418
Sham + Vehicle Ipsi #3	1388.042	1.991	697.158212
Sham + Vehicle Ipsi #4	1109.045	1.92	577.6276042
Stroke + Vehicle Contra #1	1463.272	1.995	733.4696742
Stroke + Vehicle Contra #2	1327.155	2.259	587.4966799
Stroke + Vehicle Contra #3	1659.107	2.424	684.4500825
Stroke + Vehicle Contra #4	1257.69	2.125	591.8541176
Stroke + 1mg/kg Contra #1	1441.052	2.543	566.6740071
Stroke + 1mg/kg Contra #2	1304.699	2.133	611.6732302
Stroke + 1mg/kg Contra #3	1262.233	2.458	513.5203417
Stroke + 1mg/kg Contra #4	1388.019	2.289	606.3866317
Stroke + 3mg/kg Contra #1	1587.045	2.169	731.6943292
Stroke + 3mg/kg Contra #2	1552.077	2.285	679.2459519
Stroke + 3mg/kg Contra #3	1464.712	2.281	642.1359053
Stroke + 3mg/kg Contra #6	1360.693	1.771	768.3190288
Sham + Vehicle Contra #1	661.828	1.219	542.9269893
Sham + Vehicle Contra #2	1100.212	1.865	589.9260054
Sham + Vehicle Contra #3	896.411	1.833	489.040371
Sham + Vehicle Contra #4	1086.289	1.814	598.8362734

Table 4

pTrkB levels by ELISA

Sample	OD
Stroke + Vehicle Ipsi #1	0.022
Stroke + Vehicle Ipsi #3	0.006
Stroke + Vehicle Ipsi #5	0.061
Stroke + Vehicle Ipsi #6	0.017
Stroke + 1mg/kg Ipsi #1	0.061
Stroke + 1mg/kg Ipsi #2	0.153
Stroke + 1mg/kg Ipsi #3	0.107
Stroke + 3mg/kg Ipsi #1	0.05
Stroke + 3mg/kg Ipsi #4	0.061
Stroke + 3mg/kg Ipsi #6	0.029
Sham + Vehicle Ipsi #1	0.008
Sham + Vehicle Ipsi #2	0.025
Sham + Vehicle Ipsi #3	0.013
Stroke + Vehicle Contra #1	0.061
Stroke + Vehicle Contra #3	0.094
Stroke + Vehicle Contra #5	0.144
Stroke + Vehicle Contra #6	0.163
Stroke + 1mg/kg Contra #1	0.106
Stroke + 1mg/kg Contra #2	0.095
Stroke + 1mg/kg Contra #3	0.188
Stroke + 3mg/kg Contra #1	0.073
Stroke + 3mg/kg Contra #4	0.005
Stroke + 3mg/kg Contra #6	0.009
Stroke + 3mg/kg Contra #7	0.008
Sham + Vehicle Contra #1	0.003
Sham + Vehicle Contra #2	0.103
Sham + Vehicle Contra #3	0.221

Note that the standards for the pTrkB failed repeatedly. For this reason the data are reported as raw optical density measurements
